# (*S*)-1,2-Dimethyl-1,1,2-triphenyl-2-(4-piperidiniometh­yl)disilane chloride

**DOI:** 10.1107/S1600536808028808

**Published:** 2008-09-17

**Authors:** Christian Däschlein, Viktoria H. Gessner, Carsten Strohmann

**Affiliations:** a Anorganische Chemie, Technische Universität Dortmund, Otto-Hahn-Strasse 6, 44227 Dortmund, Germany

## Abstract

The title compound, C_26_H_34_NSi_2_^+^·Cl^−^, shows chirality at silicon. Because of its highly selective synthesis with an e.r. of >99:1 by means of a racemic resolution with mandelic acid, the free disilane is of great importance to the chemistry of highly enanti­omerically enriched lithio­silanes and their trapping products. N—H⋯Cl hydrogen bonding is present between the protonated nitro­gen atom of the piperidino group and the chloride counter-anion. The silicon–silicon distance as well as silicon–carbon and carbon–nitro­gen bond lengths are in the same ranges as in other quaternary, functionalized di- and tetra­silanes.

## Related literature

For details of lithio­silanes, see: Lickiss & Smith (1995 ▶); Sekiguchi *et al.* (2000 ▶); Strohmann *et al.* (2001 ▶, 2006 ▶); Strohmann & Däschlein (2008*a* ▶,*b* ▶); Tamao & Kawachi (1995 ▶). For enanti­o­merically enriched lithio­silanes, see: Colomer & Corriu (1976 ▶); Oestreich *et al.* (2005 ▶); Omote *et al.* (2000 ▶); Sommer & Mason (1965 ▶); Strohmann *et al.* (2007 ▶). For the determination of the absolute configuration of the disilane as the mandelic acid adduct, see: Strohmann *et al.* (2002 ▶). For related literature on hydro­chlorides of amines, see: Farrugia *et al.* (2001 ▶).
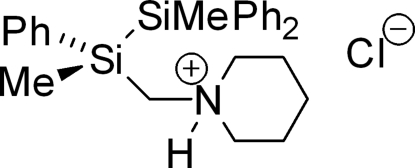


## Experimental

### 

#### Crystal data

C_26_H_34_NSi_2_^+^·Cl^−^
*M* *_r_* = 452.19
Orthorhombic, 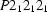

*a* = 10.120 (2) Å
*b* = 13.289 (3) Å
*c* = 18.598 (4) Å
*V* = 2501.3 (9) Å^3^
*Z* = 4
Mo *K*α radiation
μ = 0.26 mm^−1^
*T* = 173 (2) K
0.30 × 0.30 × 0.20 mm

#### Data collection

Bruker SMART APEX CCD diffractometer
Absorption correction: multi-scan (*SADABS*; Bruker, 1999 ▶) *T*_min_ = 0.926, *T*_max_ = 0.950
45451 measured reflections
4911 independent reflections
4808 reflections with *I* > 2σ(*I*)
*R*_int_ = 0.077

#### Refinement

*R*[*F* ^2^ > 2σ(*F* ^2^)] = 0.055
*wR*(*F* ^2^) = 0.149
*S* = 1.05
4911 reflections
277 parameters
H atoms treated by a mixture of independent and constrained refinement
Δρ_max_ = 0.41 e Å^−3^
Δρ_min_ = −0.34 e Å^−3^
Absolute structure: Flack (1983 ▶), 2128 Friedel pairs
Flack parameter: 0.08 (10)

### 

Data collection: *SMART* (Bruker, 2001 ▶); cell refinement: *SAINT-Plus* (Bruker, 1999 ▶); data reduction: *SAINT-Plus*; program(s) used to solve structure: *SHELXS97* (Sheldrick, 2008 ▶); program(s) used to refine structure: *SHELXL97* (Sheldrick, 2008 ▶); molecular graphics: *ORTEP-3* (Farrugia, 1997 ▶); software used to prepare material for publication: *SHELXL97*.

## Supplementary Material

Crystal structure: contains datablocks I, New_Global_Publ_Block. DOI: 10.1107/S1600536808028808/wm2191sup1.cif

Structure factors: contains datablocks I. DOI: 10.1107/S1600536808028808/wm2191Isup2.hkl

Additional supplementary materials:  crystallographic information; 3D view; checkCIF report

## Figures and Tables

**Table 1:** Hydrogen-bond geometry (Å, °)

*D*—H⋯*A*	*D*—H	H⋯*A*	*D*⋯*A*	*D*—H⋯*A*
N1—H100⋯Cl	1.00 (5)	2.05 (5)	3.031 (3)	166 (4)

**Fig. 1.**
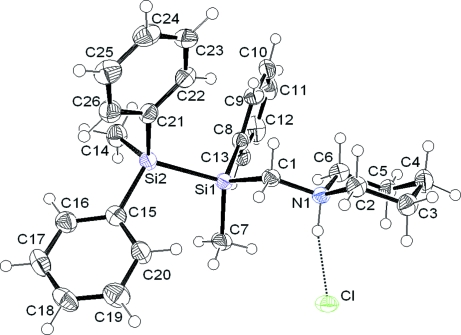
ORTEP plot of the asymmetric unit of (I) with displacement ellipsoids drawn at the 50% probability level. The dashed line indicates the hydrogen bond.

## supplementary crystallographic information

### Comment

Functionalized lithiosilanes (Strohmann *et al.*, 2001; Strohmann *et al.*, 2006; Strohmann & Däschlein, 2008a,b) are versatile reagents in organic and organometallic chemistry, e.g. for the nucleophilic introduction of protecting groups, the synthesis of silyl-substituted transition metal complexes or for silicon-based polymers (Lickiss & Smith, 1995; Sekiguchi *et al., *2000; Tamao & Kawachi, 1995*).* Especially highly enantiomerically enriched lithiosilanes are of great interest due to the increased stability of configuration at the stereogenic silicon center compared to the labile alkyllithium compounds. Yet, as the synthetic pathways to functionalized lithiosilanes are extremly limited, only six highly enantiomerically enriched systems are known until today (Colomer & Corriu, 1976; Oestreich *et al.*, 2005; Omote *et al.*, 2000; Sommer & Mason, 1965; Strohmann *et al.*, 2002; Strohmann *et al.*, 2007)*.* Thereby the Si-Si bond cleavage of aryl substituted disilanes with lithium proved to be a potential method for the preperation of these useful compounds.

(*S*)-1,2-Dimethyl-1,1,2-triphenyl-1-(piperidinomethyl)disilane, (I), is an excellent starting system for the praparation of highly enantiomerically enriched lithiosilanes as it can be synthesised in an e.r. of > 99:1 by means of a racemic resolution with mandelic acid (Strohmann *et al.*, 2002). The reaction with lithium metal results in the selective Si-Si bond cleavage and thus offers a synthetic pathway to highly enantiomerically enriched silicon-chiral di-, tri- and tetrasilanes (Strohmann *et al.*, 2007) and -germanes (Strohmann & Däschlein, 2008b).

Treatment of (I) with HCl yields the title compound, (II), (*S*)-1,2-Dimethyl-1,1,2-triphenyl-1-(piperidiniummethyl)disilane chloride, as a crystalline solid. The determination of the absolute configuration of the stereogenic silicon center gave the same absolute configuration as the mandelic acid adduct published previously (Strohmann *et al.*, 2002).

The asymmetric unit of (II) contains one molecule of the silicon-chiral disilane. Furthermore, hydrogen bonding between the hydrogen atom of the protonated nitrogen of the piperidino group and the chloride counteranion can be found (Fig. 1). The H···Cl distance (2.05 Å) and the N-H-Cl angle (166.1 °) are in the typical ranges of such hydrogen bonds (Farrugia *et al.*, 2001). With a value of 2.3672 (13) Å, the Si-Si bond length is comparable to other known systems and is slightly larger than the sum of the covalent radii of two silicon atoms (2.33 Å). The silicon-carbon and carbon-nitrogen distances, respectively, are also in the same ranges as in previously published systems. Thereby, the longest silicon-carbon distance can be found between Si1 and C1. Due to the positive charge at the nitrogen in beta-position to Si1, the bond length to C1 is increased to 1.910 (3) Å. The other five Si-C(X) bonds (X = 7, 8, 14, 15, 21) have values between 1.873 (4) and 1.884 (4) Å (average: 1.881 Å) and thus are significantly smaller than the Si1-C1 distance but in very good agreement with the sum of the covalent radii of silicon and carbon (1.88 Å). Considering the Si1-Si2-axis, it is noteworthy to mention that the substituents at the silicon atoms do possess an almost ecliptical arrangement and therefore do not adopt the sterically less hindered staggered conformation.

### Experimental

To the enantiomerically pure (*S*)-1,2-Dimethyl-1,2,2-triphenyldisilan-1-(piperidinomethyl)disilane, (I), dissolved in Et_2_O, one equivalent of etherical HCl solution was added and stored at room temperature for 24 h. After removal of the solvent, a colourless crystalline solid of (II) remained, suitable for single crystal *x*-ray studies.

^1^H-NMR (500.1 MHz, CDCl_3_): *δ* = -4.92 (s, 3H; NCSiSiC*H*_3_), -4.80 (s, 3H; NCSiC*H*_3_), 0.95–1.05, 1.55–1.60 (m, 1H each; NCCC*H*_2_), 1.40–1.50 (m, 2H; NCC*H*_2_), 1.95–2.05, 2.05–2.15 (m, 1H each; NCC*H*_2_), 2.22–2.28, 2.30–2.37 (m, 1H each; NC*H*_2_C), 2.70–2.75, 2.80–2.85 (m, 1H each; SiC*H*_2_), 3.03–3.07, 3.12–3.18 (m, 1H each; NC*H*_2_C),7.15–7.35 (m, 15H; aromat. H).

{^1^H}^13^C-NMR (125.8 MHz, CDCl_3_): *δ* = -4.9 (1 C) (NCSi*C*H_3_), -4.8 (1 C) (NCSiSi*C*H_3_), 21.3 (1 C) (NCC*C*H_2_), 22.5, 22.6 (1 C each) (NC*C*H_2_), 49.4 (1 C) (Si*C*H_2_), 55.2, 57.4 (1 C each) (N*C*H_2_C), 128.06, 128.17, 128.37 (2 C each) (all *C*-m), 129.36, 129.50,129.78 (1 C each) (all *C*-p), 134.06, 134.64,134.77 (2 C each) (all *C-*o), 133.74, 133.99,134.46 (1 C each) (all *C*-i).

{^1^H}^29^Si-NMR (99.4 MHz, CDCl_3_): *δ* = -25.3 (1Si) (NC*Si*), -23.5 (1Si) (NCSi*Si*).

### Refinement

The H atoms were refined in their ideal geometric positions using the riding model approximation with U_iso_(H) = 1.5U_eq_(C) for methyl H atoms and of U_iso_(H) = 1.2U_eq_(C) for all other H atoms except atom H100 (bonded to the N atom of the piperidino group) which was refined freely.

### Crystal data

**Table:**

C_26_H_34_NSi_2_^+^·Cl^−^	*F*(000) = 968
*M**_r_* = 452.19	*D*_x_ = 1.201 Mg m^−^^3^
Orthorhombic, *P*2_1_2_1_2_1_	Mo *K*α radiation, λ = 0.71073 Å
Hall symbol: P 2ac 2ab	Cell parameters from 999 reflections
*a* = 10.120 (2) Å	θ = 1.9–26.0°
*b* = 13.289 (3) Å	µ = 0.26 mm^−^^1^
*c* = 18.598 (4) Å	*T* = 173 K
*V* = 2501.3 (9) Å^3^	Block, colourless
*Z* = 4	0.30 × 0.30 × 0.20 mm

### Data collection

**Table:**

Bruker SMART APEX CCD diffractometer	4911 independent reflections
Radiation source: fine-focus sealed tube	4808 reflections with *I* > 2σ(*I*)
graphite	*R*_int_ = 0.077
ω–scans	θ_max_ = 26.0°, θ_min_ = 1.9°
Absorption correction: multi-scan (SADABS; Bruker, 1999)	*h* = −12→12
*T*_min_ = 0.926, *T*_max_ = 0.950	*k* = −16→16
45451 measured reflections	*l* = −22→22

### Refinement

**Table:**

Refinement on *F*^2^	Secondary atom site location: difference Fourier map
Least-squares matrix: full	Hydrogen site location: inferred from neighbouring sites
*R*[*F*^2^ > 2σ(*F*^2^)] = 0.055	H atoms treated by a mixture of independent and constrained refinement
*wR*(*F*^2^) = 0.149	*w* = 1/[σ^2^(*F*_o_^2^) + (0.0402*P*)^2^ + 1.4067*P*] where *P* = (*F*_o_^2^ + 2*F*_c_^2^)/3
*S* = 1.05	(Δ/σ)_max_ < 0.001
4911 reflections	Δρ_max_ = 0.41 e Å^−^^3^
277 parameters	Δρ_min_ = −0.34 e Å^−^^3^
0 restraints	Absolute structure: Flack (1983), 2128 Friedel pairs
Primary atom site location: structure-invariant direct methods	Flack parameter: 0.08 (10)

### Special details

**Table:**

Geometry. All e.s.d.'s (except the e.s.d. in the dihedral angle between two l.s. planes) are estimated using the full covariance matrix. The cell e.s.d.'s are taken into account individually in the estimation of e.s.d.'s in distances, angles and torsion angles; correlations between e.s.d.'s in cell parameters are only used when they are defined by crystal symmetry. An approximate (isotropic) treatment of cell e.s.d.'s is used for estimating e.s.d.'s involving l.s. planes.
Refinement. Refinement of *F*^2^ against ALL reflections. The weighted *R*-factor *wR* and goodness of fit *S* are based on *F*^2^, conventional *R*-factors *R* are based on *F*, with *F* set to zero for negative *F*^2^. The threshold expression of *F*^2^ > σ(*F*^2^) is used only for calculating *R*-factors(gt) *etc*. and is not relevant to the choice of reflections for refinement. *R*-factors based on *F*^2^ are statistically about twice as large as those based on *F*, and *R*- factors based on ALL data will be even larger.

### Fractional atomic coordinates and isotropic or equivalent isotropic displacement parameters (Å^2^)

**Table:**

	*x*	*y*	*z*	*U*_iso_*/*U*_eq_	
Cl	−0.09614 (10)	0.04149 (6)	0.25243 (5)	0.0361 (2)	
Si1	0.28622 (9)	0.21087 (7)	0.20801 (5)	0.0249 (2)	
Si2	0.40619 (9)	0.23973 (7)	0.10071 (5)	0.0276 (2)	
N1	0.0148 (3)	0.2532 (2)	0.25691 (15)	0.0246 (5)	
C3	−0.2128 (4)	0.2953 (3)	0.2978 (2)	0.0346 (8)	
H3B	−0.2411	0.2241	0.2961	0.042*	
H3A	−0.2909	0.3378	0.2876	0.042*	
C17	0.4393 (5)	0.0299 (3)	−0.0646 (2)	0.0433 (10)	
H17	0.5060	0.0075	−0.0968	0.052*	
C8	0.3754 (3)	0.2680 (3)	0.28642 (17)	0.0271 (7)	
C2	−0.1098 (3)	0.3132 (3)	0.24077 (19)	0.0309 (7)	
H2A	−0.0881	0.3858	0.2387	0.037*	
H2B	−0.1452	0.2930	0.1933	0.037*	
C16	0.4683 (4)	0.1011 (3)	−0.0121 (2)	0.0374 (9)	
H16	0.5559	0.1262	−0.0082	0.045*	
C6	0.0655 (3)	0.2780 (3)	0.32978 (17)	0.0297 (7)	
H6A	0.0922	0.3496	0.3312	0.036*	
H6B	0.1446	0.2366	0.3400	0.036*	
C1	0.1159 (3)	0.2715 (2)	0.19928 (16)	0.0273 (7)	
H1A	0.1290	0.3452	0.1956	0.033*	
H1B	0.0773	0.2489	0.1531	0.033*	
C11	0.4933 (4)	0.3529 (4)	0.4090 (2)	0.0456 (10)	
H11	0.5315	0.3812	0.4510	0.055*	
C18	0.3126 (5)	−0.0077 (3)	−0.0696 (2)	0.0435 (10)	
H18	0.2926	−0.0565	−0.1054	0.052*	
C19	0.2150 (5)	0.0243 (3)	−0.0237 (2)	0.0466 (10)	
H19	0.1282	−0.0025	−0.0273	0.056*	
C10	0.4387 (4)	0.4145 (3)	0.3574 (2)	0.0386 (9)	
H10	0.4416	0.4855	0.3630	0.046*	
C21	0.3634 (3)	0.3622 (3)	0.05534 (19)	0.0302 (7)	
C24	0.3080 (4)	0.5453 (3)	−0.0126 (2)	0.0411 (9)	
H24	0.2899	0.6077	−0.0355	0.049*	
C26	0.3724 (4)	0.3723 (3)	−0.0189 (2)	0.0366 (8)	
H26	0.3978	0.3157	−0.0469	0.044*	
C23	0.2970 (4)	0.5373 (3)	0.0615 (2)	0.0420 (9)	
H23	0.2708	0.5938	0.0894	0.050*	
C9	0.3793 (4)	0.3723 (3)	0.29742 (19)	0.0314 (7)	
H9	0.3400	0.4154	0.2627	0.038*	
C14	0.5853 (4)	0.2419 (3)	0.1259 (2)	0.0397 (8)	
H14C	0.6392	0.2482	0.0823	0.060*	
H14B	0.6081	0.1794	0.1509	0.060*	
H14A	0.6024	0.2994	0.1576	0.060*	
C7	0.2688 (4)	0.0726 (3)	0.2230 (2)	0.0357 (8)	
H7B	0.3567	0.0420	0.2266	0.054*	
H7C	0.2209	0.0426	0.1825	0.054*	
H7A	0.2198	0.0607	0.2676	0.054*	
C4	−0.1613 (4)	0.3193 (3)	0.3725 (2)	0.0394 (9)	
H4A	−0.1416	0.3920	0.3762	0.047*	
H4B	−0.2293	0.3024	0.4088	0.047*	
C22	0.3244 (4)	0.4466 (3)	0.0945 (2)	0.0363 (8)	
H22	0.3166	0.4415	0.1452	0.044*	
C12	0.4926 (4)	0.2503 (4)	0.3995 (2)	0.0435 (9)	
H12	0.5315	0.2080	0.4348	0.052*	
C5	−0.0374 (4)	0.2590 (3)	0.38669 (18)	0.0334 (8)	
H5A	−0.0012	0.2774	0.4344	0.040*	
H5B	−0.0595	0.1864	0.3876	0.040*	
C20	0.2446 (4)	0.0968 (3)	0.0282 (2)	0.0368 (9)	
H20	0.1770	0.1196	0.0596	0.044*	
C15	0.3720 (4)	0.1364 (3)	0.03482 (18)	0.0314 (8)	
C13	0.4352 (4)	0.2081 (3)	0.33837 (18)	0.0338 (8)	
H13	0.4369	0.1372	0.3321	0.041*	
C25	0.3450 (4)	0.4636 (3)	−0.0533 (2)	0.0434 (9)	
H25	0.3518	0.4690	−0.1041	0.052*	
H100	−0.007 (5)	0.180 (4)	0.254 (3)	0.050 (12)*	

### Atomic displacement parameters (Å^2^)

**Table:**

	*U*^11^	*U*^22^	*U*^33^	*U*^12^	*U*^13^	*U*^23^
Cl	0.0441 (5)	0.0229 (4)	0.0413 (5)	−0.0063 (4)	−0.0004 (4)	−0.0006 (3)
Si1	0.0270 (5)	0.0232 (4)	0.0246 (4)	−0.0006 (4)	0.0003 (4)	−0.0014 (3)
Si2	0.0272 (5)	0.0288 (4)	0.0269 (4)	0.0008 (4)	0.0010 (4)	−0.0027 (4)
N1	0.0219 (13)	0.0218 (13)	0.0300 (13)	−0.0003 (11)	−0.0012 (11)	−0.0007 (11)
C3	0.0275 (17)	0.0338 (18)	0.0425 (19)	0.0043 (15)	−0.0010 (16)	0.0033 (16)
C17	0.059 (3)	0.040 (2)	0.0312 (18)	0.018 (2)	0.0047 (17)	−0.0007 (16)
C8	0.0232 (16)	0.0319 (18)	0.0263 (14)	−0.0005 (13)	0.0004 (13)	−0.0045 (13)
C2	0.0268 (17)	0.0288 (16)	0.0370 (17)	0.0046 (14)	−0.0029 (15)	0.0047 (14)
C16	0.044 (2)	0.0319 (19)	0.036 (2)	0.0022 (16)	0.0021 (17)	0.0010 (15)
C6	0.0299 (18)	0.0297 (17)	0.0296 (15)	−0.0014 (14)	−0.0039 (13)	0.0027 (14)
C1	0.0310 (17)	0.0254 (16)	0.0254 (15)	−0.0010 (13)	0.0006 (13)	0.0002 (12)
C11	0.035 (2)	0.067 (3)	0.034 (2)	0.003 (2)	−0.0007 (17)	−0.0203 (19)
C18	0.066 (3)	0.037 (2)	0.0277 (17)	0.006 (2)	−0.0085 (19)	−0.0083 (15)
C19	0.046 (2)	0.048 (2)	0.046 (2)	0.000 (2)	−0.0119 (19)	−0.0086 (18)
C10	0.030 (2)	0.040 (2)	0.046 (2)	−0.0015 (16)	0.0077 (16)	−0.0142 (17)
C21	0.0263 (18)	0.0330 (17)	0.0314 (17)	−0.0021 (14)	0.0021 (14)	0.0005 (14)
C24	0.038 (2)	0.035 (2)	0.051 (2)	−0.0022 (17)	−0.0056 (17)	0.0141 (18)
C26	0.036 (2)	0.039 (2)	0.0353 (19)	−0.0011 (16)	−0.0011 (16)	−0.0027 (16)
C23	0.044 (2)	0.0252 (17)	0.057 (2)	−0.0022 (17)	0.0085 (19)	0.0011 (17)
C9	0.0306 (19)	0.0304 (17)	0.0333 (17)	−0.0031 (14)	−0.0030 (15)	0.0015 (15)
C14	0.0336 (19)	0.047 (2)	0.0386 (19)	0.0019 (18)	−0.0008 (16)	−0.0016 (17)
C7	0.046 (2)	0.0242 (16)	0.0369 (18)	−0.0006 (16)	0.0047 (16)	0.0003 (14)
C4	0.035 (2)	0.045 (2)	0.0382 (19)	0.0024 (17)	0.0073 (16)	−0.0065 (17)
C22	0.036 (2)	0.0367 (19)	0.0360 (18)	−0.0055 (16)	0.0053 (15)	0.0010 (16)
C12	0.038 (2)	0.061 (3)	0.0323 (18)	0.0150 (19)	−0.0073 (16)	0.0049 (19)
C5	0.0322 (18)	0.0378 (19)	0.0302 (17)	−0.0004 (15)	0.0056 (14)	−0.0009 (15)
C20	0.036 (2)	0.039 (2)	0.0353 (19)	0.0032 (16)	0.0016 (16)	−0.0080 (15)
C15	0.037 (2)	0.0297 (17)	0.0281 (17)	0.0024 (15)	−0.0028 (14)	0.0009 (13)
C13	0.034 (2)	0.0348 (18)	0.0325 (17)	0.0053 (16)	−0.0001 (14)	0.0002 (15)
C25	0.049 (2)	0.044 (2)	0.0368 (19)	−0.0060 (19)	−0.0076 (18)	0.0085 (18)

### Geometric parameters (Å, °)

**Table:**

Cl—H100	2.05 (5)	C18—H18	0.9500
Si1—C7	1.867 (4)	C19—C20	1.397 (6)
Si1—C8	1.876 (3)	C19—H19	0.9500
Si1—C1	1.910 (3)	C10—C9	1.386 (5)
Si1—Si2	2.3672 (13)	C10—H10	0.9500
Si2—C14	1.873 (4)	C21—C26	1.390 (5)
Si2—C15	1.873 (4)	C21—C22	1.393 (5)
Si2—C21	1.884 (4)	C24—C25	1.375 (6)
N1—C6	1.486 (4)	C24—C23	1.388 (6)
N1—C1	1.502 (4)	C24—H24	0.9500
N1—C2	1.521 (4)	C26—C25	1.400 (6)
N1—H100	1.00 (5)	C26—H26	0.9500
C3—C2	1.506 (5)	C23—C22	1.381 (6)
C3—C4	1.518 (5)	C23—H23	0.9500
C3—H3B	0.9900	C9—H9	0.9500
C3—H3A	0.9900	C14—H14C	0.9800
C17—C18	1.379 (7)	C14—H14B	0.9800
C17—C16	1.392 (6)	C14—H14A	0.9800
C17—H17	0.9500	C7—H7B	0.9800
C8—C13	1.391 (5)	C7—H7C	0.9800
C8—C9	1.402 (5)	C7—H7A	0.9800
C2—H2A	0.9900	C4—C5	1.511 (5)
C2—H2B	0.9900	C4—H4A	0.9900
C16—C15	1.389 (5)	C4—H4B	0.9900
C16—H16	0.9500	C22—H22	0.9500
C6—C5	1.506 (5)	C12—C13	1.395 (5)
C6—H6A	0.9900	C12—H12	0.9500
C6—H6B	0.9900	C5—H5A	0.9900
C1—H1A	0.9900	C5—H5B	0.9900
C1—H1B	0.9900	C20—C15	1.398 (5)
C11—C12	1.374 (7)	C20—H20	0.9500
C11—C10	1.377 (6)	C13—H13	0.9500
C11—H11	0.9500	C25—H25	0.9500
C18—C19	1.374 (6)	N1—Cl	3.031 (3)
			
C7—Si1—C8	109.16 (17)	C11—C10—H10	120.2
C7—Si1—C1	110.05 (17)	C9—C10—H10	120.2
C8—Si1—C1	109.24 (14)	C26—C21—C22	117.4 (3)
C7—Si1—Si2	109.48 (13)	C26—C21—Si2	120.9 (3)
C8—Si1—Si2	110.06 (11)	C22—C21—Si2	121.7 (3)
C1—Si1—Si2	108.84 (10)	C25—C24—C23	120.5 (4)
C14—Si2—C15	110.72 (17)	C25—C24—H24	119.7
C14—Si2—C21	108.73 (17)	C23—C24—H24	119.7
C15—Si2—C21	107.34 (15)	C21—C26—C25	121.6 (4)
C14—Si2—Si1	106.77 (13)	C21—C26—H26	119.2
C15—Si2—Si1	109.76 (12)	C25—C26—H26	119.2
C21—Si2—Si1	113.55 (12)	C22—C23—C24	119.4 (4)
C6—N1—C1	112.3 (2)	C22—C23—H23	120.3
C6—N1—C2	110.5 (3)	C24—C23—H23	120.3
C1—N1—C2	109.8 (2)	C10—C9—C8	122.0 (3)
C6—N1—H100	110 (3)	C10—C9—H9	119.0
C1—N1—H100	105 (3)	C8—C9—H9	119.0
C2—N1—H100	109 (3)	Si2—C14—H14C	109.5
C2—C3—C4	112.0 (3)	Si2—C14—H14B	109.5
C2—C3—H3B	109.2	H14C—C14—H14B	109.5
C4—C3—H3B	109.2	Si2—C14—H14A	109.5
C2—C3—H3A	109.2	H14C—C14—H14A	109.5
C4—C3—H3A	109.2	H14B—C14—H14A	109.5
H3B—C3—H3A	107.9	Si1—C7—H7B	109.5
C18—C17—C16	119.4 (4)	Si1—C7—H7C	109.5
C18—C17—H17	120.3	H7B—C7—H7C	109.5
C16—C17—H17	120.3	Si1—C7—H7A	109.5
C13—C8—C9	116.9 (3)	H7B—C7—H7A	109.5
C13—C8—Si1	121.2 (3)	H7C—C7—H7A	109.5
C9—C8—Si1	121.8 (3)	C5—C4—C3	109.5 (3)
C3—C2—N1	110.6 (3)	C5—C4—H4A	109.8
C3—C2—H2A	109.5	C3—C4—H4A	109.8
N1—C2—H2A	109.5	C5—C4—H4B	109.8
C3—C2—H2B	109.5	C3—C4—H4B	109.8
N1—C2—H2B	109.5	H4A—C4—H4B	108.2
H2A—C2—H2B	108.1	C23—C22—C21	121.9 (4)
C15—C16—C17	121.5 (4)	C23—C22—H22	119.1
C15—C16—H16	119.3	C21—C22—H22	119.1
C17—C16—H16	119.3	C11—C12—C13	120.4 (4)
N1—C6—C5	111.4 (3)	C11—C12—H12	119.8
N1—C6—H6A	109.3	C13—C12—H12	119.8
C5—C6—H6A	109.3	C6—C5—C4	111.2 (3)
N1—C6—H6B	109.3	C6—C5—H5A	109.4
C5—C6—H6B	109.3	C4—C5—H5A	109.4
H6A—C6—H6B	108.0	C6—C5—H5B	109.4
N1—C1—Si1	119.1 (2)	C4—C5—H5B	109.4
N1—C1—H1A	107.5	H5A—C5—H5B	108.0
Si1—C1—H1A	107.5	C19—C20—C15	121.2 (4)
N1—C1—H1B	107.5	C19—C20—H20	119.4
Si1—C1—H1B	107.5	C15—C20—H20	119.4
H1A—C1—H1B	107.0	C16—C15—C20	117.7 (3)
C12—C11—C10	119.9 (4)	C16—C15—Si2	121.9 (3)
C12—C11—H11	120.1	C20—C15—Si2	120.3 (3)
C10—C11—H11	120.1	C8—C13—C12	121.1 (4)
C19—C18—C17	121.0 (4)	C8—C13—H13	119.4
C19—C18—H18	119.5	C12—C13—H13	119.4
C17—C18—H18	119.5	C24—C25—C26	119.1 (4)
C18—C19—C20	119.3 (4)	C24—C25—H25	120.4
C18—C19—H19	120.4	C26—C25—H25	120.4
C20—C19—H19	120.4	N1—H100—Cl	166.1 (41)
C11—C10—C9	119.6 (4)		

### Hydrogen-bond geometry (Å, °)

**Table:**

*D*—H···*A*	*D*—H	H···*A*	*D*···*A*	*D*—H···*A*
N1—H100···Cl	1.00 (5)	2.05 (5)	3.031 (3)	166 (4)
